# Peer Review of “Offenders With Personality Disorder Who Fail to Progress: A Case-Control Study Using Partial Least Squares Structural Equation Modeling Path Analysis”

**DOI:** 10.2196/33935

**Published:** 2021-10-29

*This is a peer-review report submitted for the paper “Offenders With Personality Disorder Who Fail to Progress: A Case-Control Study Using Partial Least Squares Structural Equation Modeling Path Analysis”.*

## Round 1 Review

### General Comments

This study [1] identified and empirically investigated the causal drivers behind nonprogression of offenders with personality disorder on the UK Offender Personality Disorder pathway. Interestingly, the study found negative attitudes toward treatment to be the leading driver of nonprogression, closely followed by psychopathology. Overall, I found this to be a very interesting and potentially highly impactful paper, with specific practical and applied value. My suggestions for improvement are mostly minor and primarily surround a general neatening-up of the writing and presentation of the manuscript.

### Major Comments

1. The Introduction is interesting to read, easy to follow, and well structured. I think the only thing that it is missing is some more background information on personality disorders. This could involve simply defining personality disorder in a general sense and providing some information surrounding risks associated with them in community populations as well as their prevalence rates in offender populations (with citations). I would probably put this information at the start of the Introduction before going into more specific detail surrounding offenders with personality disorder. Some language/grammatical improvements are also required throughout the introduction.
2. The “Procedure” section in the Methods seems unnecessary as its own section. I would consider incorporating this information in the “Sample” section, potentially changing the heading to “Sample and Procedure.”
3. In the Results section, I would suggest adding the test statistics and *P* values to Table 1, as this would make it very easy to identify where there were key differences.
4. The numbers and interpretation of the results in Table 1 are somewhat difficult to follow. Percentagewise, a higher proportion of the nonprogression group are single compared to the control group, but the chi-square test results indicate that the control group is more likely to be single than the nonprogression group (presumably based on the actual N). I think this needs to be clarified/made consistent in the manuscript.
5. I think that it would be informative for the reader for the authors to merge all the supplementary descriptive results tables (Tables S1-S4) into one table and incorporate this into the main text in the Results section (rather than supplemental materials), also adding the test statistics to the tables.
6. The Discussion section was particularly interesting to read. Given the potential clinical impact and practical nature of this study, the only thing I think the Discussion is missing is a section on clinical/practical implications of the study (which was very briefly touched upon in the conclusion). I think that this would really benefit the manuscript and be of interest to readers.

### Minor Comments

1. This is very minor, but the “Engagement With Treatment and Treatment Noncompletion” in the Introduction could probably just be “Engagement With Treatment,” as this covers treatment noncompletion.
2. I would avoid using the term “personality disordered” throughout the manuscript.
3. The semicolons in the first paragraph of the “Engagement With Treatment and Treatment Noncompletion” section should be colons (and in some other parts of the manuscript).
4. Some of the study objectives in the Introduction could be made somewhat more specific/precise (eg, explicitly stating “offenders with personality disorder,” rather than just offenders, or offenders that have not progressed on the Offenders Personality Disorder pathway).
5. The capitalization of “N” within tables needs to be made consistent throughout.
6. When referring to tables in the manuscript, the “t” should be capitalized throughout (eg, “Table 2”).
7. In the Results section, there may in fact be too much detail regarding the structural model assessment; for example, there is no need to explain what each of the parameters mean/represent.
8. *P* values should be reported as exact numbers (ie, to 3 decimal points), and *P* values of *P*=.000 should be reported as *P*<.001.
